# Convalescent Home, Saltburn-By-The-Sea

**Published:** 1888-03-17

**Authors:** Elizabeth Cox

**Affiliations:** Matron. Convalescent Home, Saltburn-by-the-Sea


### THE EDITOR'S LETTER-BOX.

[Our Correspondents are reminded that prolixity is a great bar to publication, and that brevity of style and conciseness of statement greatly facilitate early insertion.]

### CONVALESCENT HOME, SALTBURN-BY-THE-SEA.

I HAVE seen in your admirable paper notices of convalescent homes, and I think I would like you to know of this one. Twenty years last August the Pease family, of Darlington (Quakers), opened a convalescent home in two cottages, one for men and one for women, taking ten of each sex. After five years they erected the present building for fifty, afterwards increasing the number of beds to 64. The total cost of the building and furnishing was £12,000. During these twenty years the Pease family have entirely maintained the cost, each patient coming free. The time allowed—three weeks—in some cases has extended to six or eight weeks. The home was intended for Messrs. Pease's own workpeople, but patients from all parts have been taken. We have also taken Bible-women, nurses, and ladies of reduced income into a private room, also free. The applications have, however, been so numerous that the Messrs. Pease have decided to make a change, regarding which I enclose a circular.

ELIZABETH COX, Matron.
Convalescent Home, Saltburn-by-the-Sea.

